# Near infrared absorption/emission perylenebisimide fluorophores with geometry relaxation-induced large Stokes shift†

**DOI:** 10.1039/d0ra07050e

**Published:** 2020-09-30

**Authors:** Jie Ma, Yizhi Zhang, Hongbo Zhang, Xifeng He

**Affiliations:** Heilongjiang Provincial Key Laboratory of Catalytic Synthesis for Fine Chemicals, College of Chemistry and Chemical Engineering, Qiqihar University Qiqihar 161006 P. R. China majie722@126.com

## Abstract

The dyes (P-1 and P-2) of perylenebisimide (PBI) conjugated with 2-(2-hydroxyphenyl)benzothiazole (HBT) were prepared by Sonogashira coupling reaction. The new compounds have special photophysical properties, such as near infrared absorption/emission and large Stokes shift. The UV-vis absorption (range from 651 nm to 690 nm) and emission wavelength (range from 732 nm to 756 nm) of P-1 and P-2 extend to near infrared range. Importantly, they have much larger Stokes shifts (range from 73 nm to 105 nm) compared with the conventional PBI derivatives, such as 7 (from 19 nm to 65 nm) and 9 (from 81 nm to 86 nm). TD-DFT calculation was used to rationalize UV-vis absorption, emission and especially large Stokes shift from the theoretical point of view. We found geometry relaxation of P-1 and P-2 in the excited state is an important reason for the origin of large Stokes shift besides intramolecular electron transfer (ICT).

## Abstract 2

The dyes with near infrared absorption/emission and large stokes shifts induced by geometry relaxation were prepared.

## Graphical abstract

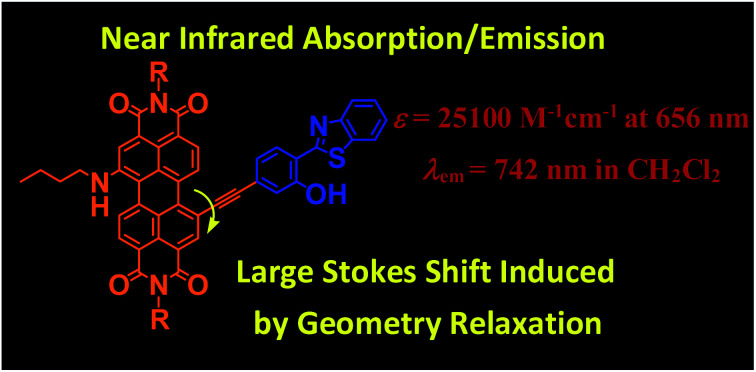

## Introduction

Recently, perylenebisimide (PBI) and its derivatives have been widely applied to the areas of fluorescence probe and intracellular fluorescence imaging image,^1–5^ organic photovoltaic devices,^6–12^ charge transporting materials,^13–18^ luminescent materials^19–24^ and triplet photosensitizers^25–32^ due to its excellent luminous performance, photo-stability and electronic transmission capacity. Traditional PBI dyes always show the strong absorption in visible region and with small Stokes shift (generally less than 60 nm).^15,33^ The above drawbacks are detrimental to the applications such as fluorescent molecular probes and intracellular fluorescence imaging due to its weak tissue penetrating ability. Therefore, PBI dyes with near infrared absorption/emission, as well as large Stoke shift are very meaningful.

On the other hand, HBT is a conventional dye with excited intramolecular proton transfer (ESIPT),^34,35^ which has been applied to fluorescent molecular probes^36–49^ and luminescent materials.^50,51^ By connecting PBI with 2-(2-hydroxyphenyl)benzothiazole (HBT), π-conjugation structure is enlarged by the introduction of substituents in PBI, so the absorption wavelength may be extended to near infrared region. Meanwhile, with the increase of the substituents in PBI core, geometry distortion will be obvious in the ground state. Upon photoexcitation, the geometry relaxation maybe occurs in the excited state, thus the large Stokes shift is obtained. To the best of our knowledge, PBI units containing ESIPT chromophores have never been reported.

In order to address the above challenges, we designed and prepared two new dyes (P-1 and P-2) based on PBI conjugated HBT by acetylene bond. Compare with the parent compound (7 and 9), the most remarkable photophysical properties of P-1 and P-2 are with near infrared absorption/emission and large Stokes shifts (73–105 nm). Our work will provide a new way to obtain such special dyes.

## Results and discussion

### Molecular design and synthesis of PBI derivatives

**Scheme 1:**
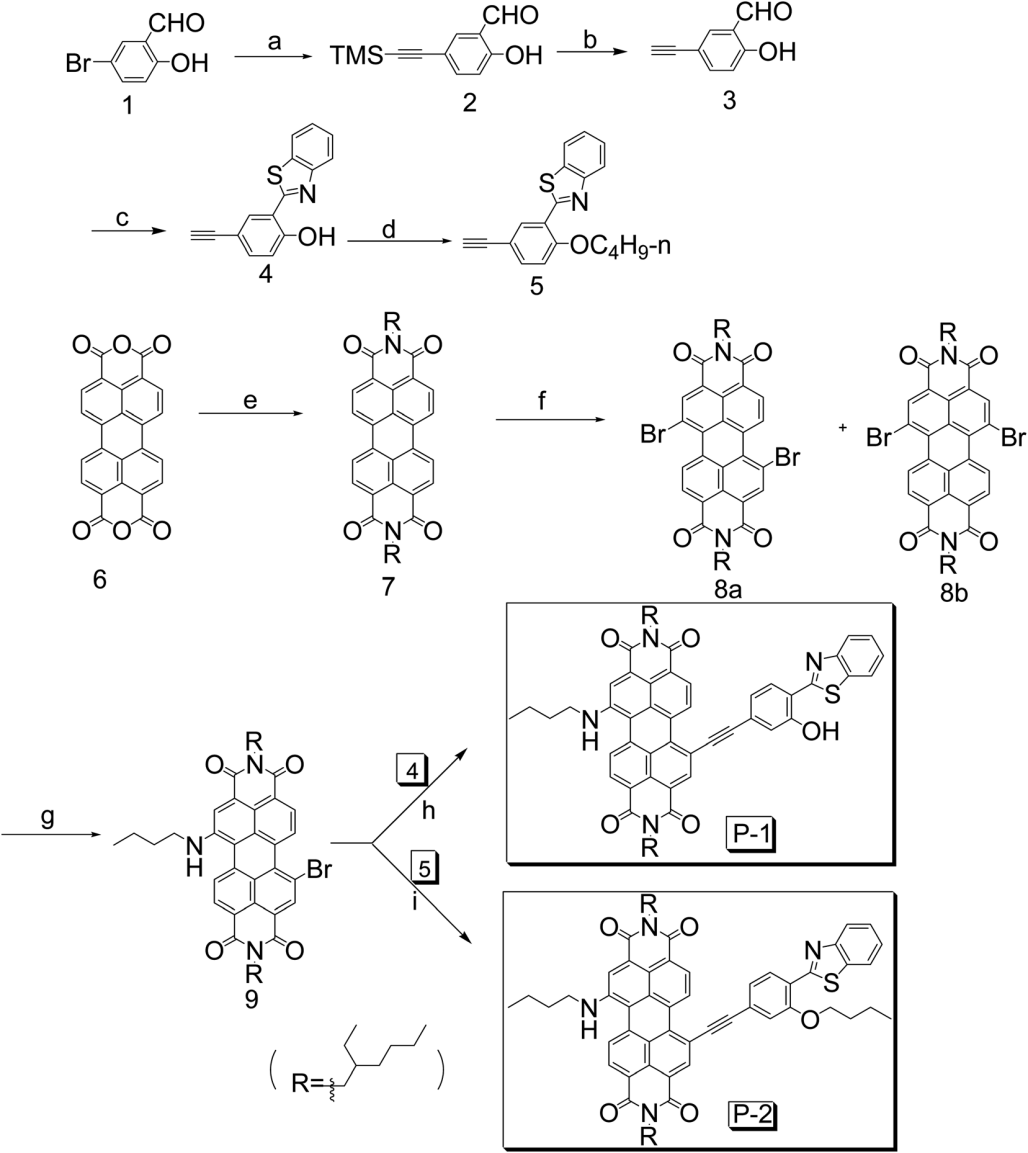
Synthesis of compounds P-1, P-2, 7 and 9. (a) Trimethylsilylacetylene, Pd(PPh_3_)_2_Cl_2_, CuI, PPh_3_, NEt_3_, reflux, 6 h; (b) TBAF, THF; (c) 2-aminothiophenol, MeOH; (d) *n*-C_4_H_9_Br, K_2_CO_3_, DMF; (e) 2-ethyl hexyl amine, imidazole; (f) Br_2_, CHCl_3_ reflux, 48 h; (g) butylamine, 25 °C, 8 h; (h) Pd(PPh_3_)_4_, CuI, NEt_3_, reflux, 10 h; (i) Pd(PPh_3_)_4_, CuI, NEt_3_, reflux, 10 h.

It is well-known that introducing electron-donating substituents in the bay-position of PBI can extend the absorption wavelength efficiently.^52–54^ Herein we introduce alkylamino group and HBT respectively in PBI moiety to get longer absorption wavelength. P-1 and P-2 were prepared according to the synthetic route (Scheme 1). PBI (compound 7) undergoes bromination and amination reaction (compound 9) successively in bay-position to attain longer wavelength. The key procedure is Sonogashira coupling reaction catalyzed by Pd (0) to get the target compounds P-1 and P-2. Interestingly, we found that PBI without alkylamino substitution cannot carry out the coupling reaction. It is due to alkylamino group make up the electron deficiency in PBI core. In order to compare the photophysical property with P-1, we synthesized P-2 which is alkylation of the hydroxyl group in HBT. Compounds 7 and 9 are employed as reference compounds to testify the origin of large Stokes shift. All the compounds were obtained in moderate to satisfying yields (see Experimental section and ESI† for details).

### Uv-vis absorption and fluorescence emission spectrums

**Fig. 1:**
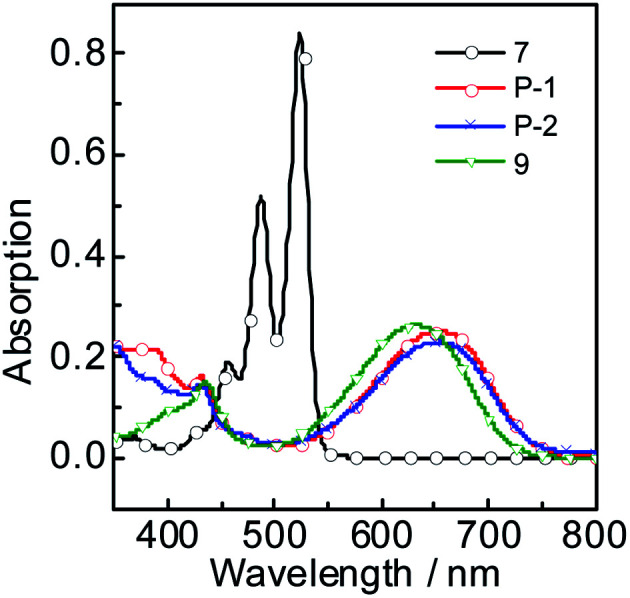
UV-vis absorption of 7, P-1, P-2 and 9 in DCM, 1.0 × 10^−5^ M, 20 °C.

The UV-vis absorption of P-1, P-2, 7 and 9 were studied (Fig. 1). P-1 shows the absorption wavelength at 656 nm (*ε* = 25 100 M^−1^ cm^−1^) in dichloromethane (Fig. 1). P-2 gives the similar absorption at 658 nm and a bit lower absorption (*ε* = 22 900 M^−1^ cm^−1^) was observed (Fig. 1). Compared with P-1 and P-2, the reference compound 7 shows the two absorption maxima at 487 nm and 524 nm and it is S_0_ → S_1_ transition with clear vibronic fine structure.^55^ Compound 9 give the absorption at 634 nm. As a parent compounds, 7 has the strongest absorption. Once the bay-positions of PBI are substituted, the absorption decreases dramatically, such as P-1, P-2 and 9, which is due to the twisting of PBI core. Especially the absorption maximum of P-1 and P-2 both extend to near infrared range (up to 656 nm). With the increase of polarity of solvent, all the compounds give minor changes in the absorption wavelength, which indicates the weak electron interaction exists at the ground state.

**Fig. 2:**
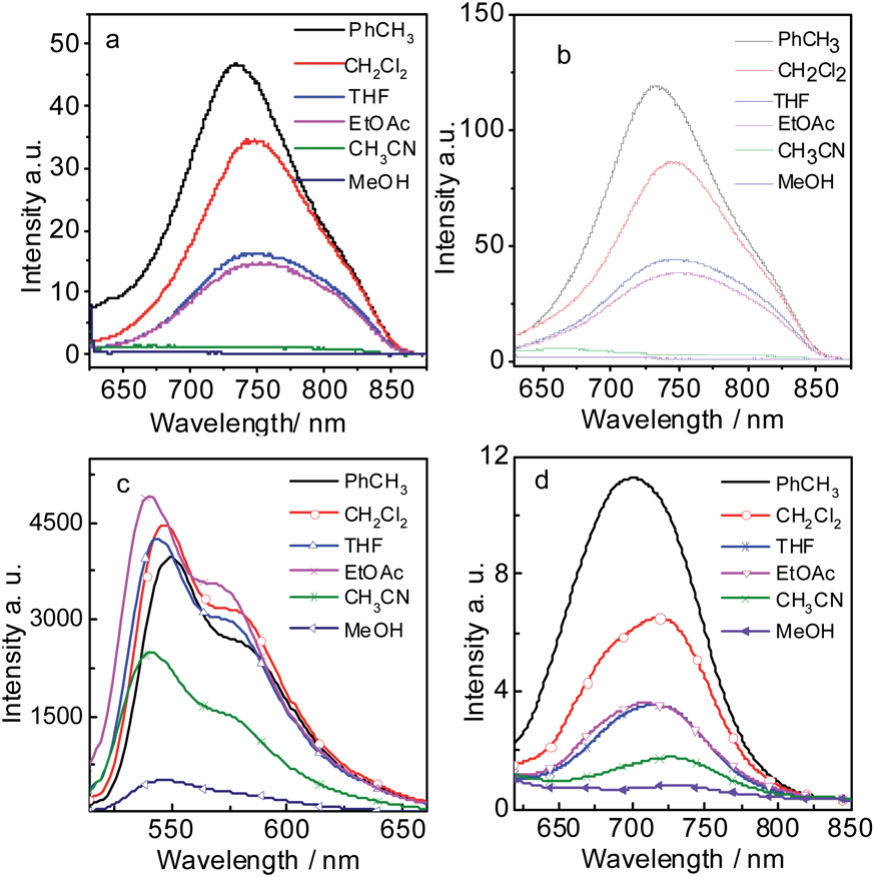
Fluorescence emission spectra of (a) P-1 (*λ*_ex_ = 620 nm), (b) P-2 (*λ*_ex_ = 620 nm), (c) 7 (*λ*_ex_ = 500 nm) and (d) 9 (*λ*_ex_ = 600 nm) in different solutions. *c* = 1.0 × 10^−5^ M, 20 °C.

The fluorescence emission spectrum of all the compounds were studied in different solvents (Fig. 2). Compound 7 gives strong fluorescence emission at 546 nm (*Φ*_F_ = 99.2%) in dichloromethane (Fig. 2c). For compound 9, the emission wavelength extend to 720 nm and the emissive intensity drop significantly (Fig. 2d). P-1 shows the emission maximum at 742 nm (*Φ*_F_ = 0.48%) in dichloromethane and the emission intensity deceases gradually in the polar solvent (Fig. 2a and Table 1). P-2 gives the similar emission at 744 nm (*Φ*_F_ = 0.44%). The emissions of P-1 and P-2 are quenched completely in MeOH and CH_3_CN, which means intramolecular charge transfer (ICT) exists in P-1 and P-2. However, the fluorescence lifetime of P-1 shows less changes in PhCH_3_ (*τ*_F_ = 1.55 ns) and DCM (*τ*_F_ = 1.45 ns). The same result was also observed in P-2 (Table 1). It suggested ICT is not dramatic for such molecules which can be supported by the emission wavelength of P-1 and P-2 showing the minor changes in different solvents (Tables 1 and S1†). The likely reason is that ICT effect is weakened by torsion in PBI core brought by the substituents (see the later DFT calculation section). The low fluorescence quantum yields are due to PBI core is twisted into two naphthalimide planes A and B, so the conjugated structure is diminished. In order to testify no aggregation in P-1 and P-2, the UV-vis absorption/emission spectrums were carried out at different concentration (Fig. S15†). The linear dependence of absorbance *vs.* concentration of UV-vis absorption spectrum was observed.

**Table 1:** Photophysical properties of 7, P-1, P-2, and 9^a^

Comp.	Solvent	*λ*_abs_ b (nm)/*A*^f^	*ε* c (M^−1^ cm^−1^)	*λ*_em_ d (nm)	*τ* e (ns)	*Φ*_F_ g %	Stokes shift
P-1	PhCH_3_	651/0.227	22 700	733	1.55	0.69	82
	DCM	656/0.251	25 100	742	1.45	0.48	86
	THF	658/0.229	22 900	750	—h	0.30	92
P-2	PhCH_3_	659/0.214	21 400	732	1.75	0.61	73
	DCM	658/0.229	22 900	744	1.75	0.44	86
	THF	657/0.227	22 700	748	—h	0.25	91
7	PhCH_3_	526/0.742	74 200	545	—h	98.3	19
	DCM	524/0.869	86 900	546	—h	99.2	22
	THF	520/0.811	81 100	544	—h	98.8	24
9	PhCH_3_	624/0.231	23 100	702	—i	—i	78
	DCM	634/0.262	26 200	720	—i	—i	86
	THF	636/0.229	22 900	716	—i	—i	80

a The excited wavelength for compounds P-1, P-2, 7 and 9 were 620 nm, 620 nm, 500 nm and 600 nm respectively (1.0 × 10^−5^ M, 20 °C).

b Absorption wavelength.

c Molar extinction coefficient.

d Fluorescence emission wavelength.

e Fluorescence lifetimes.

f Absorbance.

g Fluorescence quantum yields with compound MB (*Φ* = 0.03 in MeOH) as the standard for P-1 and P-2 and with acridine yellow (*Φ* = 0.57 in MeOH) as the standard for 7.

h Not determined.

i Weak signal.

The Stokes shifts of P-1 and P-2 range from 73 nm to 105 nm in different solvents (Tables 1 and S1†). For example, the Stokes shifts of P-1 and P-2 is 86 nm in DCM (Table 1), which is larger than the conventional PBI derivatives.^15,55,56^ Compared with P-1 and P-2, 7 has much smaller Stokes shift (from 19 nm to 65 nm). The Stokes shifts of 9 range from 78 nm to 86 nm, which is a bit smaller than P-1 and P-2. The reason will be presented in the later DFT calculation section.

Meanwhile, we found that the emission wavelength of P-1 and P-2 does not vary significantly with the polarity of different solvents, which indicates the large Stokes is not completely attributed to solvent-polarity induced phenomenon. This result means that the large Stokes shift of the new PBI derivatives is partially due to intrinsic property.^56^ Interestingly, although P-1 has HBT moiety, it has not the characteristic of ESIPT, but still with large Stokes shifts. Especially in the weak polarity solvent, the Stokes shifts of P-1 and P-2 are 82 nm and 73 nm in PhCH_3_ respectively (Table 1). It indicates that some internal structural feature result in the large Stoke shift besides ICT effect^57^(see the later DFT calculation section). Large Stokes shift is necessary for the fluorophores because it can effectively reduce self-absorption or the inner filter effect.^56^

### DFT calculation to rationalize the absorption/emission and large Stokes shifts

DFT calculations have been widely utilized to the study of fluorophores and fluorescent molecular probes.^58–61^ Herein we used DFT calculation to rationalize photophysical properties, such as absorption, emission and the origin of large Stokes shift.

Generally Stokes shift is affected by π-conjugation structure and ICT effect induced by the polarities of the solvents. Another main factor is the geometry relaxation between the S_0_ state and the first singlet state (S_1_ state).^62–64^ According to Frank–Condon principle, the geometry does not change from S_0_ (ground state) to S_1_ (Frank–Condon state) upon photoexcitation. If the geometry relaxation occurs between S_1_ state (Frank–Condon state) and S_1_ state (fluorescence emissive state), the large Stokes shift will generated. Hence we optimized S_0_ and S_1_ state of all the compounds and employed toluene as the calculated solvent.

**Fig. 3:**
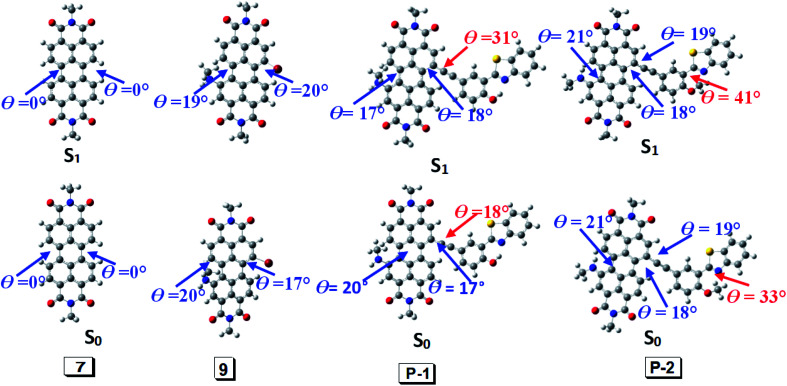
Geometry of 7, 9, P-1 and P-2 at the ground state (S_0_) and the singlet excited state (S_1_). Toluene was used as solvent in the calculation. Calculated at B3LYP/6-31g(d) level with Gaussian 09W.

According to DFT result, we found compound 7 has no geometry changes at S_0_ and S_1_ state (Fig. 3), which is in accordance with experimental result of the smaller Stokes shifts (Tables 1 and S1†). For P-1, the ground state geometry changes dramatically compared with 7. For example, the dihedral of PBI moiety change from 0° (in 7) to 20° (in P-1), thus the non-coplanarity of P-1 is much obvious than 7 in the ground state. Interestingly, the dihedral of PBI moiety have no changes from Frank–Condon state to S_1_ state (fluorescence emissive state) in P-1, but the obvious variation is observed between PBI and HBT moieties (the dihedral increases from 18° to 31°). The variation in P-1 is attributed to the big steric hindrance effect between PBI and HBT units in Frank–Condon state, which is released by geometry relaxation in S_1_ state. Therefore, the large Stokes shift is obtained for P-1.

Different from P-1, the geometry variation of P-2 does not generate between PBI and HBT units. We found the dihedral between benzene ring and benzothiazole increases from 33° to 41°, while the angle in P-1 is 0° (Fig. 3). The steric hindrance produced by alkylation of hydroxyl group is probably responsible for P-2 geometry relaxation in S_1_ state.

9 has large Stokes shift but it is a bit smaller than P-1 and P-2. Geometry relaxation is not responsible for the large Stokes shift because only minor change appears from Frank–Condon state to S_1_ state (fluorescence emissive state) (Fig. 3). Hence it can be speculated its large Stoke shift is attributed to intramolecular electron transfer (ICT).

**Fig. 4:**
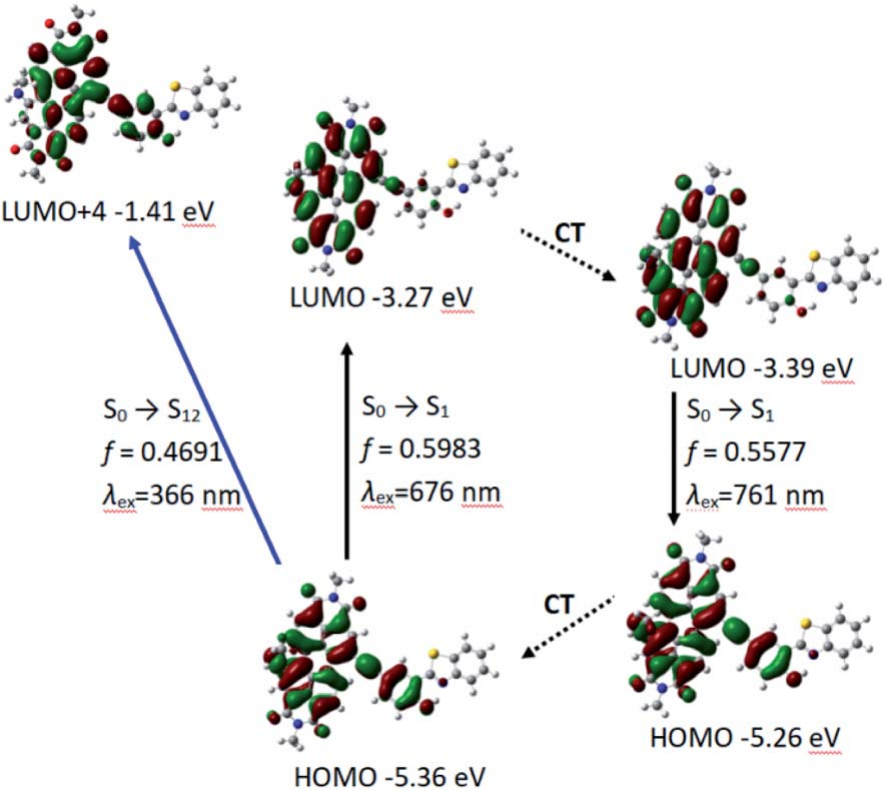
The frontier MOs involved in the vertical excitation and the emission of P-1. CT stands for conformation transformation. Calculated with TDDFT method based on the optimized ground state (S_0_) and the lowest-lying singlet excited state (S_1_) geometry. Toluene was used as solvent in the calculations. Calculated at B3LYP/6-31g(d) level with Gaussian 09W.

We also carried out the time-dependent DFT (TDDFT) calculations for UV-vis absorption and fluorescence emission of P-1 and P-2. For P-1, the calculated UV-vis absorption is 676 nm (oscillator strength is 0.5983) which is close to the experimental value of 651 nm. Meanwhile the calculated fluorescence emission wavelength is 761 nm which is also near to the experimental wavelength of 733 nm (Table 2 and Fig. 4).

**Table 2:** Selected parameters for the vertical excitation (UV-vis absorption and fluorescence emission) of the compounds. Electronic excitation energies (eV) and oscillator strengths (*f*), configurations of the low-lying excited states of P-1 and its fluorescent precursors. Calculated by TDDFT//B3LYP/6-31G(d), based on the optimized ground state geometries (PhCH_3_ was employed as solvent in all the calculation)

	Electronic transition^a^	Excitation energy	TDDFT/B3LYP/6-31G(d)		
			*f* b	Composition^c^	CI^d^
Absorption	S_0_ → S_1_	1.83 eV (676 nm)	0.5983	H → L	0.7066
	S_0_ → S_3_	2.87 eV (432 nm)	0.1901	H − 2 → L	0.6467
				H − 3 → L	0.2316
	S_0_ → S_12_	3.39 eV (366 nm)	0.4691	H → L + 4	0.4344
Emission	S_1_ → S_0_	1.63 eV (761 nm)	0.5577	H → L	0.7080

a Only selected excited states were considered. The numbers in parentheses are the excitation energy in wavelength.

b Oscillator strength.

c H stands for HOMO and L stands for LUMO. Only the main configurations are presented.

d Coefficient of the wavefunction for each excitations. The CI coefficients are in absolute values.

PBI and phenylacetylene units contribute to HOMO, while LUMO is more localized on PBI moiety, which indicates intramolecular electron transfer occurs to some extent, but it is not very obvious (Fig. 4). The result is in consistent with the experimental results of UV-vis absorption spectra, fluorescence spectra and fluorescent lifetime (Fig. 2a and Table 1). Meanwhile, P-1 has HBT moiety but it is absent of ESIPT. From electron distribution of HOMO and LUMO (S_1_ state and Frank–Condon state), it does not spread to nitrogen atom, so the basicity of N-atom is very weak in the excited state. That is not beneficial for ESIPT.

**Fig. 5:**
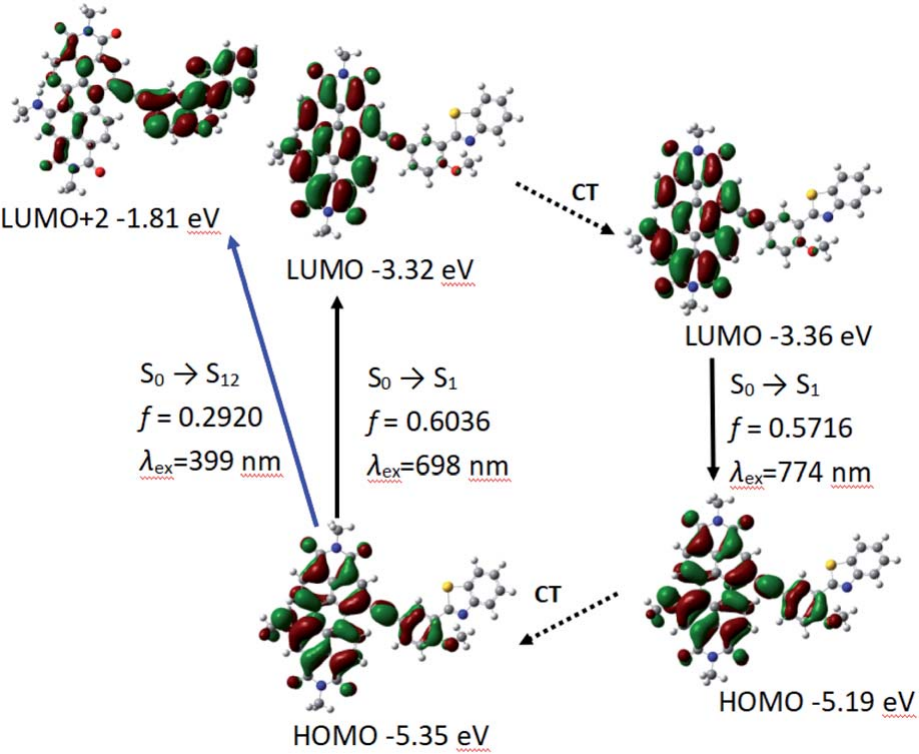
The frontier MOs involved in the vertical excitation and the emission of P-2. CT stands for conformation transformation. Calculated with TDDFT method based on the optimized ground state (S_0_) and the lowest-lying singlet excited state (S_1_) geometry. Toluene was used as solvent in the calculations. Calculated at B3LYP/6-31g(d) level with Gaussian 09W.

Similar to P-1, PBI and phenylacetylene bond moieties in P-2 contributes significantly to HOMO, but only PBI and acetylene bond contributes to LUMO. It indicates that ICT is not very obvious in P-2, either. The calculated absorption and fluorescence emission wavelength are 698 nm and the oscillator strength is 0.6036, which is close to the experimental result of 659 nm. The calculated emission of 774 nm is in good agreement to the experimental results of 732 nm (Table 3 and Fig. 5).

**Table 3:** Selected parameters for the vertical excitation (UV-vis absorption and fluorescence emission) of the compounds. Electronic excitation energies (eV) and oscillator strengths (*f*), configurations of the low-lying excited states of P-2 and its fluorescent precursors. Calculated by TDDFT//B3LYP/6-31G(d), based on the optimized ground state geometries (PhCH_3_ was employed as solvent in all the calculation)

	Electronic transition^a^	Excitation energy	TDDFT/B3LYP/6-31G(d)		
			*f* b	Composition^c^	CI^d^
Absorption	S_0_ → S_1_	1.78 eV (698 nm)	0.6036	H → L	0.7079
	S_0_ → S_7_	3.11 eV (399 nm)	0.2920	H → L + 2	0.5506
				H → L + 3	0.4283
	S_0_ → S_12_	3.36 eV (369 nm)	0.1451	H − 8 → L	0.5258
				H → L + 4	0.3169
Emission	S_1_ → S_0_	1.60 eV (774 nm)	0.5716	H → L	0.7099

a Only selected excited states were considered. The numbers in parentheses are the excitation energy in wavelength.

b Oscillator strength.

c H stands for HOMO and L stands for LUMO. Only the main configurations are presented.

d Coefficient of the wavefunction for each excitations. The CI coefficients are in absolute values.

### Redox properties: cyclic voltammogram of P-1 and P-2

The electrochemical data of P-1 and P-2 are summarized in Table 4. For P-1, there are two reversible reductive waves observed at −1.04 V and −1.24 V (Table 4 and Fig. S16†), which are similar to the reported PBI derivatives.^55,65^ A reversible oxidation wave at +0.69 V which can be attributed to the HBT moiety, because PBI has no oxidative potential. Similar results are also observed in P-2. According to the electrochemical data, the energy levels of HOMO and LUMO of P-1 were calculated as −5.13 eV and −3.40 eV, which is near to DFT calculated results (−5.36 eV and 3.27 eV) (Fig. 4). The energy gap between HOMO and LUMO is 1.73 eV which is close to DFT calculation result of 2.01 eV. The similar results were obtained for P-2.

**Table 4:** Electrochemical data of P-1 and P-2^a^

Compd	Oxidation (V)	Reduction (V)		*E*_HOMO_ b (eV)	*E*_LUMO_ c (eV)	*E*_g_ d (eV)
	I	I	II			
P-1	+0.69	−1.04	−1.24	−5.13	−3.40	1.73
P-2	+0.70	−1.04	−1.24	−5.14	−3.40	1.74

a Recorded with [Bu_4_N][PF_6_] as the electrolyte in CH_2_Cl_2_ (0.1 M) at ambient temperature with a scan rate of 50 mV s^−1^. Potentials are expressed as the half-wave potentials (*E*_1/2_) in volts *vs.* SCE using ferrocene as an internal reference.

b Calculated from empirical formula: *E*_HOMO_ = −(*E*_ox_ + 4.44 eV).

c Calculated by *E*_LUMO_ = −(*E*_red_ + 4.44 eV).

d Determined by *E*_g_ = HOMO − LUMO.^55^

## Conclusions

In summary, the new dyes of PBI conjugated HBT (P-1 and P-2) were prepared. Compared with traditional PBI compound (compounds 7 and 9), they show the near infrared absorption (651–690 nm) and fluorescence emission (732–756 nm). Especially, they have large Stokes shifts in polar and weak-polar solvents (73–105 nm). It is a meaningful property for only one electron-donating substitute (alkylamino group) in PBI core. We used TDDFT calculation to rationalize Uv-vis absorption, fluorescence emission and especially for the large Stokes shift. The origin of large Stokes shift of P-1 and P-2 is mainly attributed to the geometry relaxation between the S_1_ state (Frank–Condon state) and S_1_ state (fluorescence emission state). While the large Stokes shift (81–86 nm) for reference compound 9 is mainly due to ICT. Our results are useful for the preparation of PBI dyes with NIR absorption/emission and large Stokes shift. The study of PBI derivatives with high fluorescence quantum yield in aqueous medium and large Stokes shift is under way.

## Experimental

### Analytical measurements and reagents

NMR spectra were recorded by a Bruker 600 MHz spectrometer with CDCl_3_ as solvent. High-resolution mass spectra (HRMS) were measured in a MALDI-HR MS system (UK). The absorption spectra were recorded on UV2550 UV-vis spectrophotometer (Shimadzu, Japan) and Agilent 8453 UV-vis near-IR spectrophotometer. Fluorescence spectra were measured on a RF5301 PC spectrofluorometer (Shimadzu, Japan). Fluorescence lifetimes were recorded with OB920 luminescence lifetime spectrometer (Edinburgh Instruments, UK).The reagents were analytically pure and the solvents were dried and distilled. For the preparation of the intermediate compounds, please refer to the ESI.† The new compounds (P-1 and P-2) and the intermediates were characterized by ^1^H NMR, ^13^C NMR and HRMS.

### Synthetic procedures

#### Compound 8

Under Ar atmosphere, compound 7 (2.4 g, 3.9 mmol) and bromine (9 mL, 175.7 mmol) was dissolved in chloroform. Then the mixture was stirred at 60 °C for 48 h. The mixture was washed with Na_2_SO_3_ saturated solution. The organic layer was dried over anhydrous Na_2_SO_4_ and then evaporated the solvent under reduced pressure. The residue was purified by column chromatography (silica gel, MeOH/CH_2_Cl_2_, 1 : 200, v/v) to give red solid (365 mg, yield: 13.5%). The product was obtained as a mixture of 8a and 8b. The ratio of 8a and 8b is about 82 : 18 by 600 MHz ^1^H NMR analysis and they can't be separated by column chromatography.^52^^1^H NMR (400 MHz, CDCl_3_): *δ* 9.49 (s, 1H), 8.93 (s, 1H), 8.71 (d, 1H, *J* = 3.6 Hz), 4.20–4.11 (m, 2H), 1.98–1.94 (m, 1H), 1.42–1.38 (m, 4H), 1.33–1.31 (m, 4H), 0.89–0.97 (m, 6H).

#### Compound 9

Under Ar, the mixture of compound 8 (200 mg, 0.26 mmol) and 10 mL *n*-butylamine was stirred at room temperature for 8 h. The solvent was removed under reduced pressure then the residue was purified by column chromatography (silica gel, CH_2_Cl_2_) to give the product as dark green solid. Yield: 218 mg, 75.8%.^1^ H NMR (600 MHz, CDCl_3_) *δ* 9.42 (d, 1H, *J* = 7.2 Hz), 8.87 (d, 2H, *J* = 7.2 Hz), 8.61 (d, 1H, *J* = 8.4 Hz), 8.44 (d, 1H, *J* = 7.8 Hz), 8.26 (s, 1H), 4.19–4.11 (m, 4H), 3.51 (t, 2H, *J* = 7.2 Hz), 1.98–1.94 (m, 2H), 1.82–1.77 (m, 2H),1.43–1.31 (m, 18H), 0.88–1.05 (m, 15H).

#### Compound P-1

Under argon atmosphere, Pd(PPh_3_)_4_ (11.6 mg, 0.01 mmol), CuI (1.9 mg, 0.01 mmol), TEA (4 mL, 2.80 mmol) and 9 (139.0 mg, 0.18 mmol) were added to a solution of 4 (46.0 mg, 0.18 mmol) in anhydrous THF (5 mL). The mixture was stirred at 75 °C for 10 h. The resulting solution was cooled to rt, removed the solvent under reduced pressure and the compound was purified by column chromatography for three times and TLC for one time (silica gel, CH_2_Cl_2_ as the eluent) to give the pure product as dark green solid. Yield: 50.0 mg, 36.5%. ^1^H NMR (400 MHz, CDCl_3_): *δ* 12.88 (s, 1H), 9.52 (d, 1H, *J* = 7.8 Hz), 8.89 (d, 1H, *J* = 7.8 Hz), 8.40 (d, 1H, *J* = 7.8 Hz), 8.30 (d, 1H, *J* = 7.8 Hz), 8.24 (s, 1H), 8.21 (s, 1H), 7.98 (t, 2H, *J* = 7.8 Hz), 7.56 (t, 2H, *J* = 7.2 Hz), 7.46 (t, 1H, *J* = 7.2 Hz), 7.22 (d, 1H, *J* = 9.6 Hz), 7.07 (d, 1H, *J* = 8.4 Hz), 6.76 (s, 1H), 3.90–4.15 (m, 4H), 3.66–3.63 (m, 2H), 2.05–2.01 (m, 2H), 1.94–1.89 (m, 1H), 1.84–1.80 (m, 1H), 1.73–1.67 (m, 2H), 1.41–1.25 (m, 16H), 1.15 (t, 3H, *J* = 7.2 Hz), 0.98–0.86 (m, 12H). ^13^C NMR (150 MHz, CDCl_3_): *δ* 167.79, 163.54, 163.35, 163.27, 162.68, 158.57, 151.43, 147.90, 136.59, 135.91, 132.54, 131.25, 127.21, 125.82, 125.29, 124.34, 121.67, 118.50, 118.20, 114.42, 95.31, 90.09, 44.70, 44.13, 44.04, 38.02, 37.95, 31.34, 30.85, 30.77, 28.73, 28.68, 24.14, 24.13, 23.98, 23.18, 23.10, 20.76, 14.16, 14.03, 10.58. MALDI-HRMS: *m*/*z* calcd for [C_59_H_58_N_4_O_5_–H]^−^ 934.4122; found: 934.4117.

#### Compound P-2

P-2 was obtained according to the procedure similar to that of P-1, except 5 (36.2 mg, 0.10 mmol) was used instead of 4. The pure product was obtained by column chromatography for three times and TLC for one time (silica gel, CH_2_Cl_2_ as the eluent). Dark green solid. Yield: 37.5 mg, 37.9%. ^1^H NMR (400 MHz, CDCl_3_): *δ* 9.67 (d, 1H, *J* = 7.8 Hz), 8.94 (d, 1H, *J* = 7.8 Hz), 8.55 (s, 1H), 8.44 (d, 1H, *J* = 7.8 Hz), 8.32–8.22 (m, 4H), 8.00 (d, 1H, *J* = 7.8 Hz), 7.55 (t, 1H, *J* = 7.2 Hz), 7.43 (t, 1H, *J* = 7.2 Hz), 7.35 (d, 1H, *J* = 8.4 Hz), 7.00 (d, 1H, *J* = 8.4 Hz), 6.72 (s, 1H), 4.25 (t, 2H, *J* = 6.6 Hz), 4.17–4.08 (m, 2H), 3.98–3.88 (m, 2H), 3.65–3.62 (m, 2H), 2.09–2.05 (m, 2H), 2.02–1.98 (m, 1H), 1.96–1.92 (m, 1H), 1.73–1.64 (m, 4H), 1.44–1.25 (m, 16H), 1.14–1.10 (m, 6H), 0.98–0.87 (m, 12H). ^13^C NMR (150 MHz, CDCl_3_): *δ* 163.59, 163.01, 161.18, 156.94, 152.14, 147.99, 136.29, 126.00, 124.77, 124.38, 123.41, 123.07, 121.80, 121.18, 114.62, 95.69, 90.19, 69.45, 44.64, 44.19, 43.84, 37.99, 31.29, 30.77, 29.85, 28.79, 24.11, 23.17, 20.73, 19.62, 14.16, 13.93, 10.60. MALDI-HRMS: *m*/*z* calcd for [C_63_H_66_N_4_O_5_S–H]^−^ 990.4748; found: 990.4787.

## Conflicts of interest

There are no conflicts to declare.

## Supplementary Material

RA-010-D0RA07050E-s001
