# Supplementary material for: Near infrared absorption/emission perylenebisimide fluorophores with geometry relaxation-induced large Stokes shift
Source: RSC Adv. 2020 Sep 30;10(59):35840–7. doi: 10.1039/d0ra07050e (PMC9056887; doi:10.1039/d0ra07050e)
Supplement: RA-010-D0RA07050E-s001 [file RA-010-D0RA07050E-s001.pdf]

Electronic Supplementary Material (ESI) for RSC Advances.
This journal is © The Royal Society of Chemistry 2020

Electronic Supplementary Information for:

Near infrared absorption/emission Perylenebisimide fluorophores with geometry relaxation-induced large Stokes shift

Jie Ma,*[a] Yizhi Zhang,[a] Hongbo Zhang[a] and Xifeng He [a]

Heilongjiang Provincial Key Laboratory of Catalytic Synthesis for Fine Chemicals, College of Chemistry and Chemical Engineering, Qiqihar University, Qiqihar 161006, P.R.China

E-mail: majie722@126.com

Index

1. Synthesis and molecular structure characterization data……………………………………S2-9

2. Photophysical Properties of **P-1**,**P-2** ,**7** and **9**…………………..……..……………………..S9-10

3. Cyclic Voltammogram of **P-1** and **P-2**…………………...…………..………………………...S11

4. DFT calculation results……………………………..…………..………………………..S11-16

1. Synthesis and molecular structure characterization data

synthesis of compound HBT, 2 and 3.

The synthetic procedures of **2** and **3** were according to the reference methods.[1, 2]

2: ^{1}H NMR (600 MHz, $CDCl_3$): δ 11.11 (s, 1H), 9.85 (s, 1H), 7.71 (d, 1H, *J* = 2.0 Hz), 7.61 (m, 1H), 6.94 (d, 1H, *J* = 8.6 Hz), 0.25 (s, 9H). **3**: ^{1}H NMR (600 MHz, $CDCl_3$): δ 11.11 (s, 1H), 9.87 (s, 1H), 7.73 (d, 1H, *J* = 1.8 Hz), 7.63 (m, 1H), 6.97 (d, 1H, *J* = 8.4 Hz), 3.04 (s, 1H).

Synthesis of compound 4.

2 (100 mg, 0.69 mmol) and 2-aminothiophenol (170 mg, 1.30 mmol) were mixed in methanol (10 mL) and stirred for 6 h at 30 °C. Methanol was evaporated under reduced pressure and the residue was purified by column chromatography (silica gel, petroleum /CH_2Cl_2, 3:1, v / v) to give the product as white solid. Yield: 62.2 mg, 36.0 %. ^{1}H NMR (400 MHz, $CDCl_3$): δ 8.02 (d, 1H, *J* = 8.4 Hz), 7.95 (d, 1H, *J* = 8.0 Hz), 7.86 (s, 1H), 7.55-7.43 (m, 3H), 7.08 (d, 1H, *J* = 8.4 Hz), 3.07 (s, 1H). TOF HRMS EI+ ($[C_{15}H_9NOS]^+$): calcd 251.0405, found 251.0408.

Synthesis of compound 5.

The main paragraph text follows directly on here.The mixture of compound **3** (100 mg, 0.40 mmol), K_2CO_3 (100 mg, 0.72 mmol) and dimethyl formamide (0.50 mL) were stirred at room temperature, then n-butyl bromine (244 mg, 1.80 mmol) was added. The mixture were stirred at 50 °C for 4 h, then the resulting solution was dropwised into ice water and extracted with CH_2Cl_2. The organic layer was washed with water for six times and then the organic layer was dried over anhydrous sodium sulfate. The solvent was removed under reduced pressure then the compound was purified by column chromatography (silica gel, petroleum : CH_2Cl_2, 1 : 1, v / v) to give the product as white solid. (100 mg, yield: 81.4%). ^{1}H NMR (400 MHz, $CDCl_3/CD_3OD$): δ 8.73 (s, 1H), 8.11 (d, 1H, *J* = 8.0 Hz), 7.95 (d, 1H, *J* = 8.0 Hz), 7.56 (d, 1H, *J* = 8.8 Hz), 7.52 (t, 1H, J_1 = 7.2 Hz, J_2 = 8.0 Hz), 7.40 (t, 1H, J_1 = 8.0 Hz, J_2 = 7.2 Hz), 7.00 (d, 1H, *J* = 8.8 Hz), 4.22 (m, 2H), 3.04 (s, 1H), 1.99–2.04 (m, 2H), 1.61–1.66 (m, 2H), 1.06 (t, 3H, *J* = 7.2 Hz). TOF HRMS EI+ ($[C_{19}H_{17}NOS]^+$): calcd 307.1031, found 307.1031.

Synthesis of compound 7.

The mixture of **6** (2.0 g, 5.1 mmol), imidazole (10.0 g, 146.9 mmol), and 2-ethylhexylamine (3.8 mL, 21.0 mmol) was stirred under argon atmosphere at 160 °C for 6 h. The mixture was filtered under reduced pressure then the solid was washed with a large amount of water and EtOH. The solid was dried to give dark red solid (2.4 g, yield: 77.0 %). The product was used to next step without further purification.

1. P. Yang, J. Zhao, W. Wu, X.Yu, and Y. Liu, *J. Org. Chem.,* 2012, **77**, 6166–6178.

2. J. Ma, J. Zhao, P. Yang, D. Huang, C. Zhang and Q. Li, *Chem. Commun.,* 2012, **48**, 9720–9722.

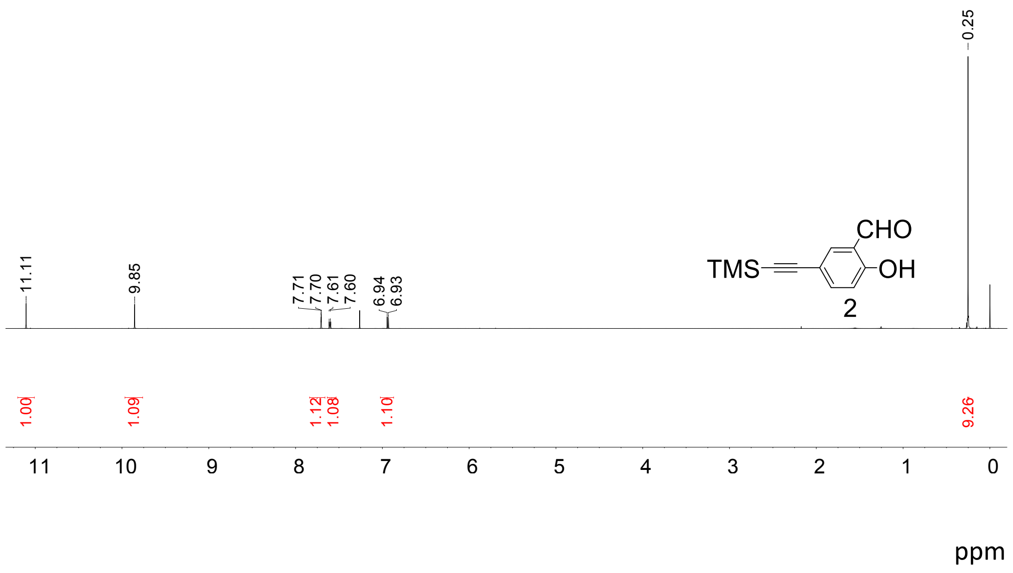

Fig S1. ^{1}H NMR of compound **2** ($CDCl_3$, 600 MHz).

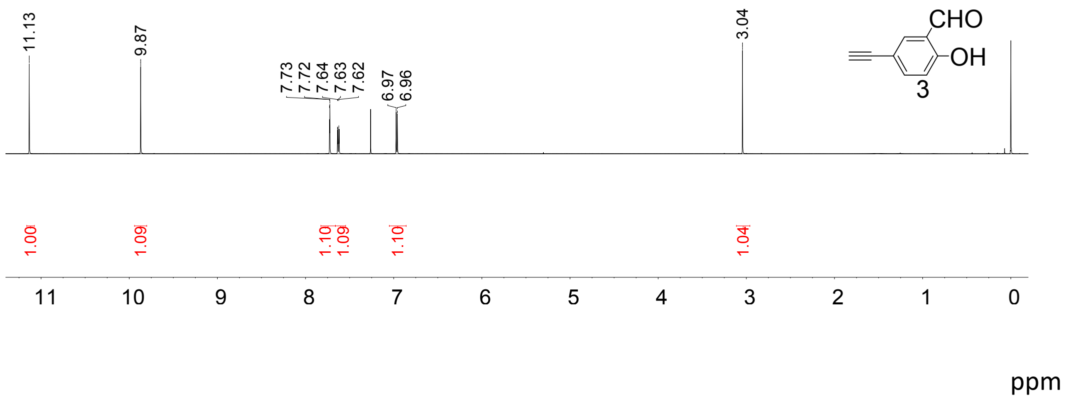

Fig S2. ^{1}H NMR of compound **3** ($CDCl_3$, 600 MHz).

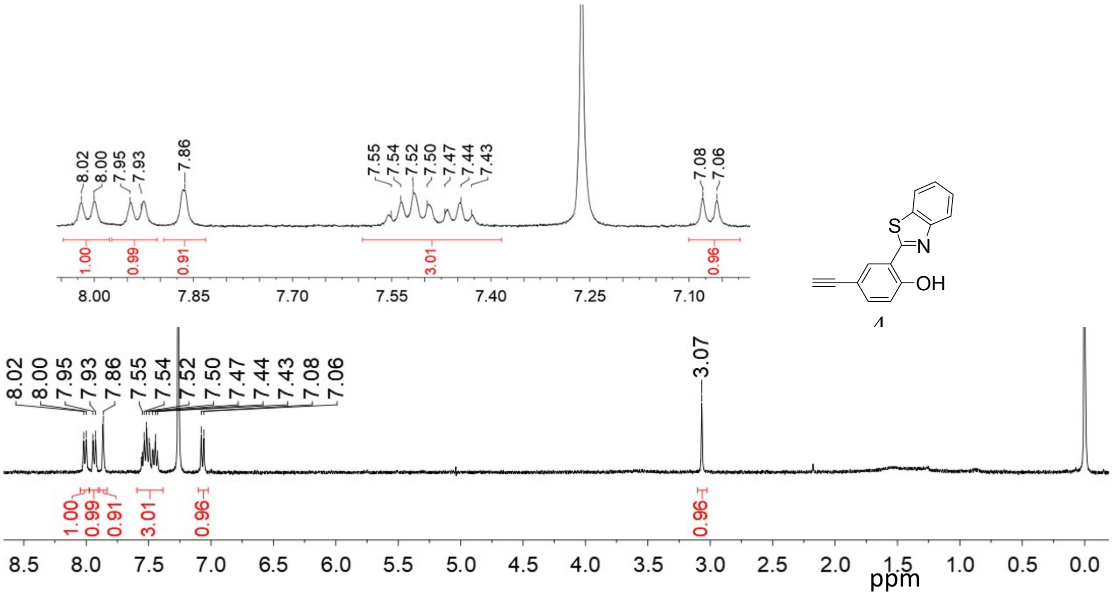

Fig S3. ^{1}H NMR of compound **4** ($CDCl_3$, 400 MHz).

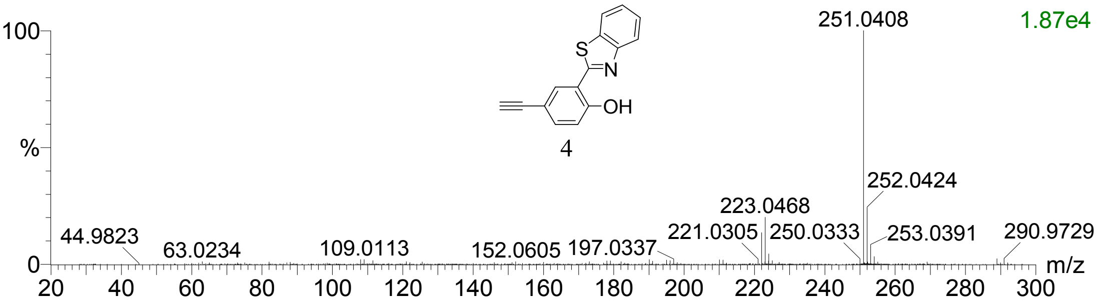

Fig S4. TOF HRMS EI^{+} of compound **4**.

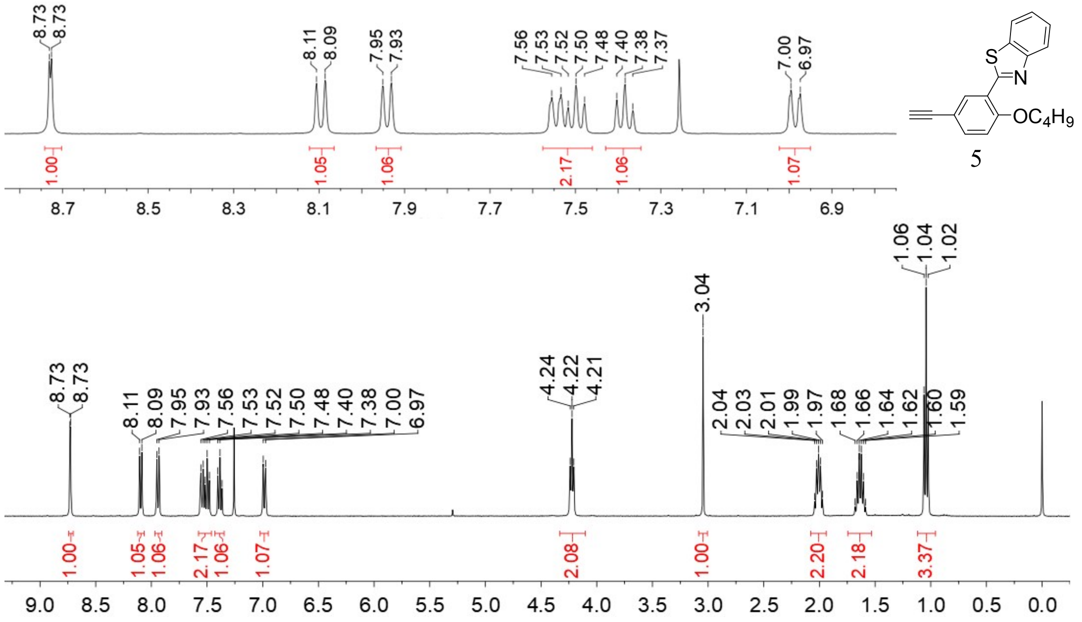

ppm

Fig S5. ^{1}H NMR of compound **5** ($CDCl_3$, 400 MHz).

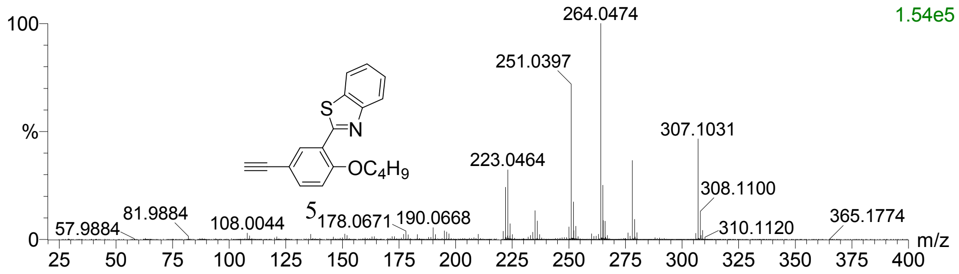

Fig S6. TOF HRMS EI+ of compound **5**

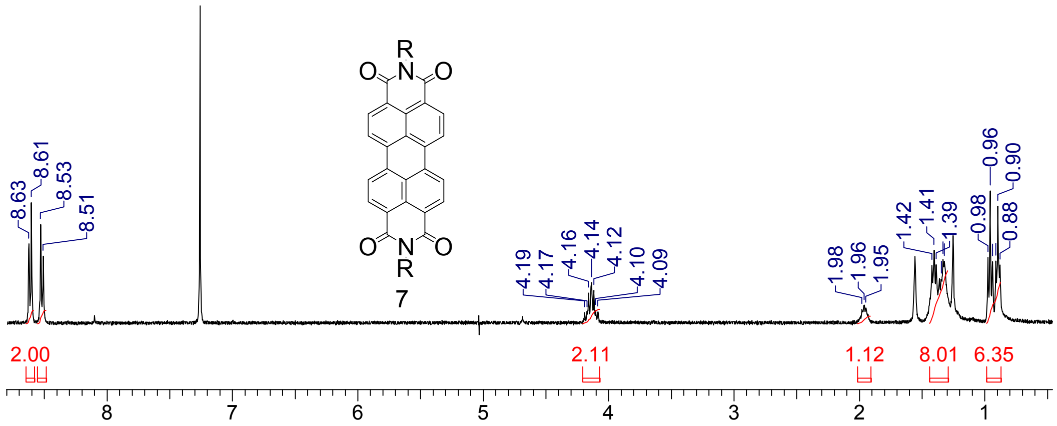

Fig S7. ^{1}H NMR of compound **7** ($CDCl_3$, 600 MHz).

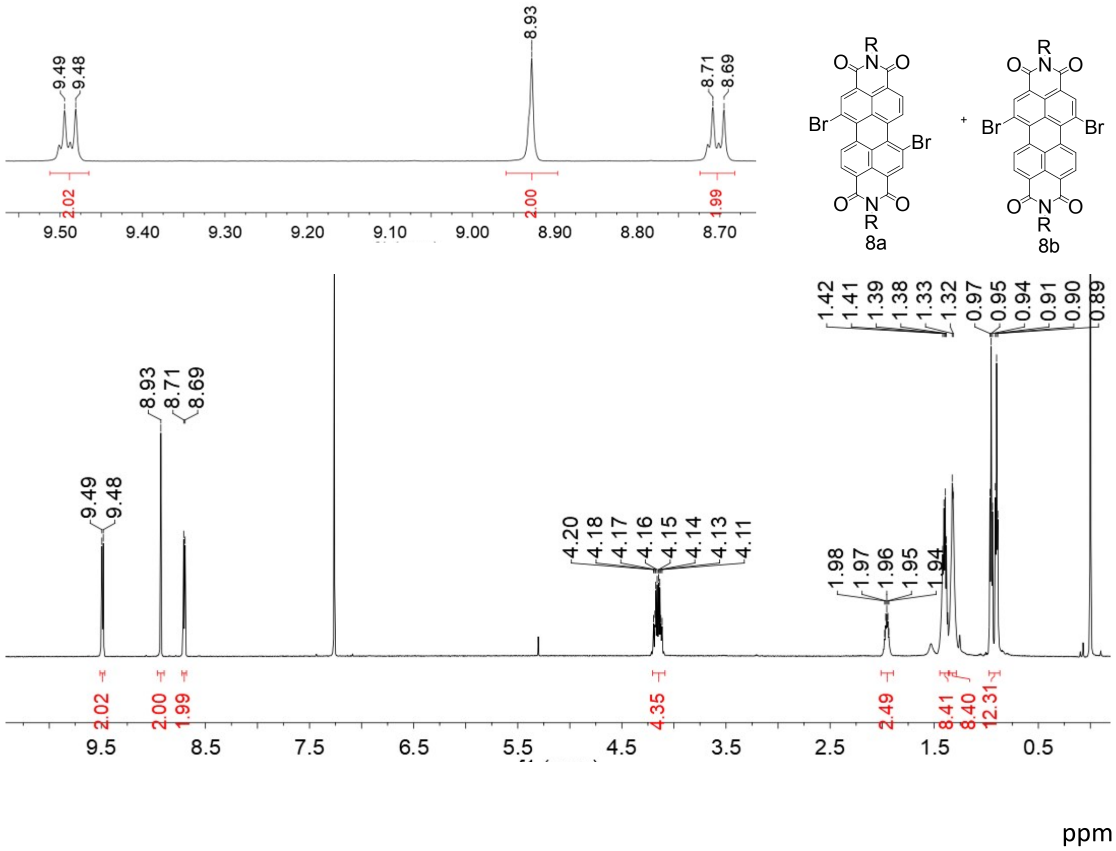

Fig S7. ^{1}H NMR of compound **8** ($CDCl_3$, 600 MHz).

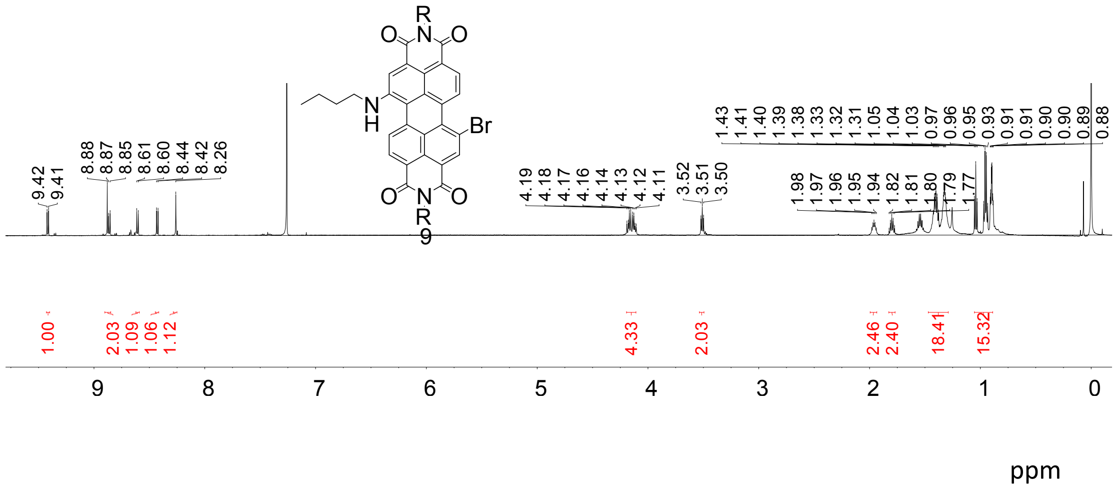

Fig S8. ^{1}H NMR of compound **9** ($CDCl_3$, 600 MHz).

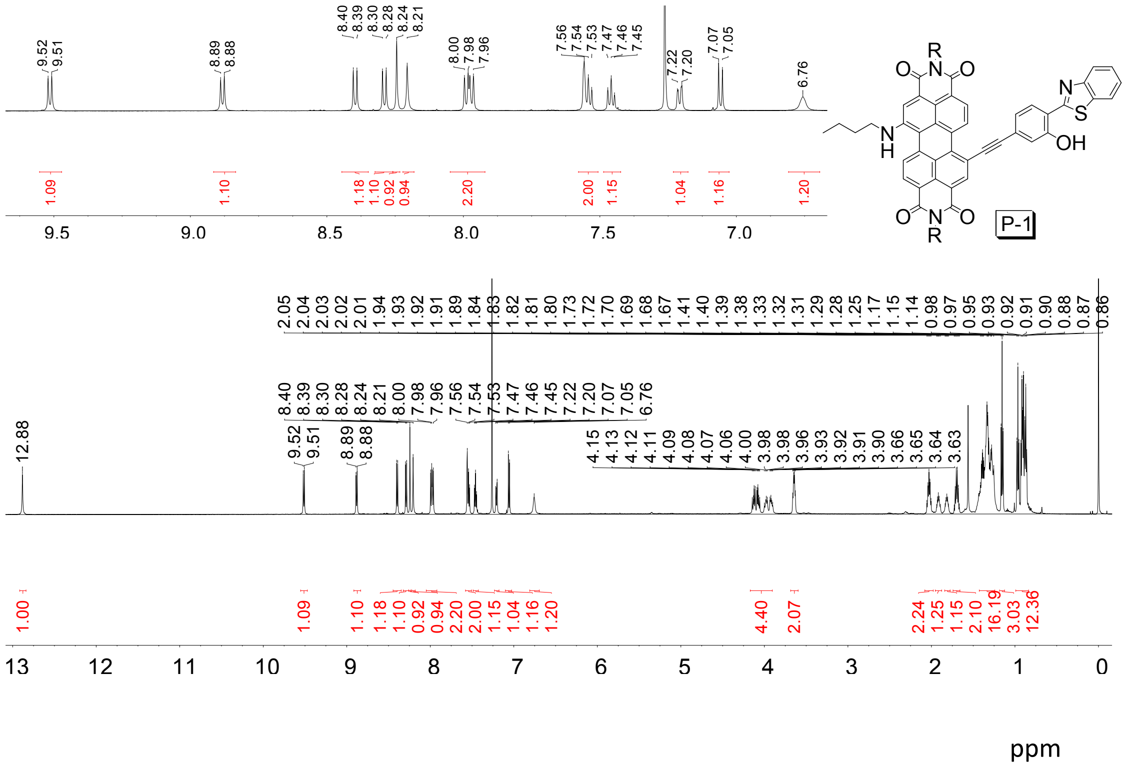

Fig S9. ^{1}H NMR of compound **P-1** ($CDCl_3$, 600 MHz).

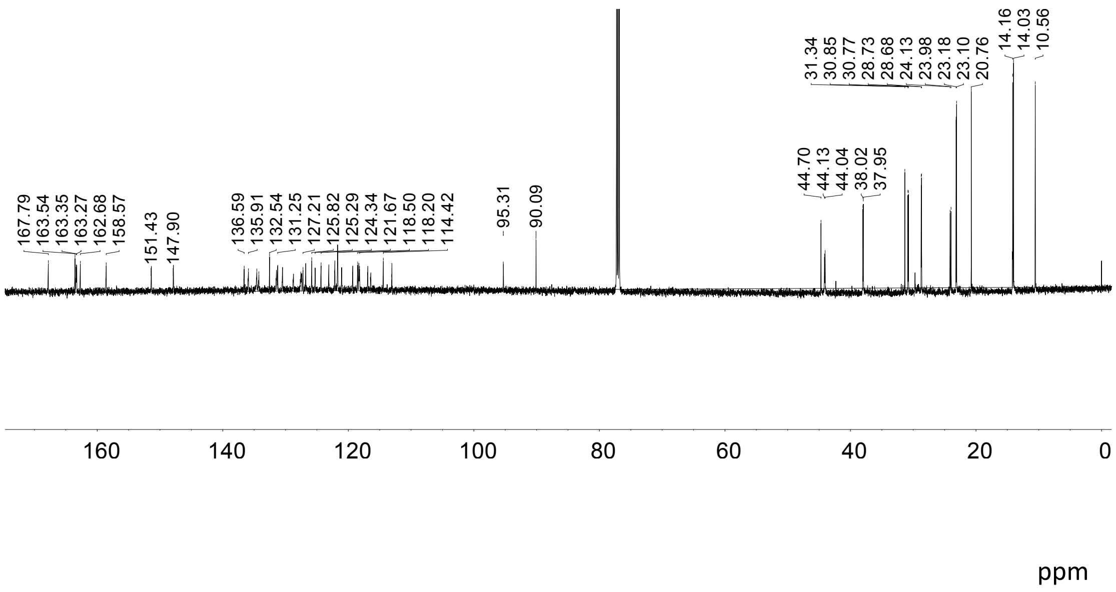

Fig S10. ^{13}C NMR of compound **P-1** ($CDCl_3$, 150 MHz).

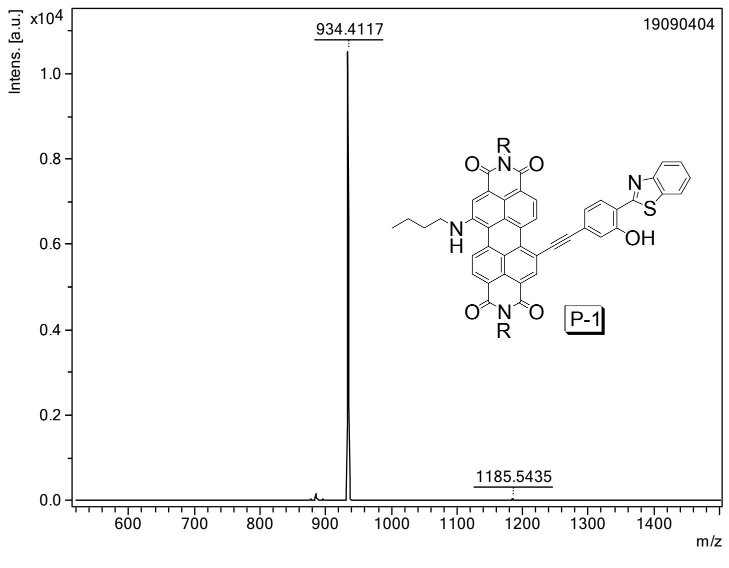

Fig S11. MALDI-HRMS of compound **P-1**

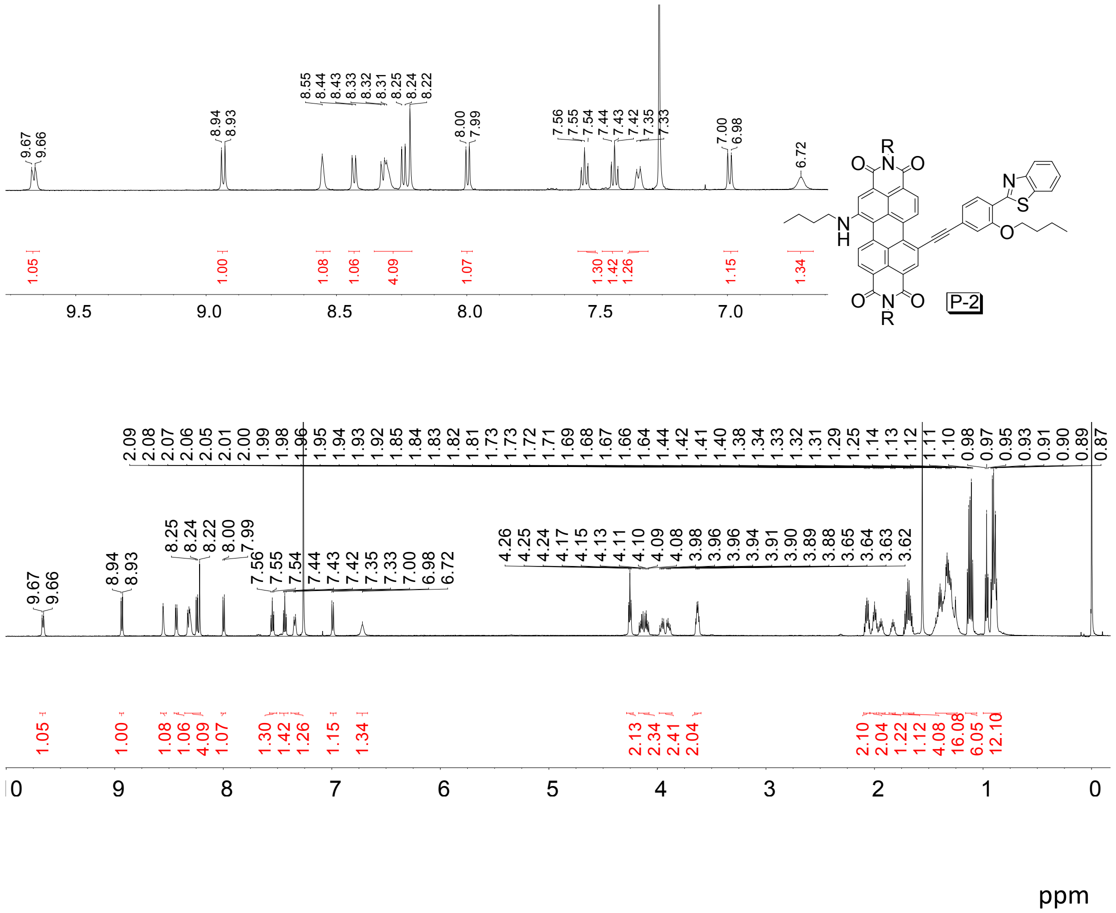

Fig S12. ^{1}H NMR of compound **P-2** ($CDCl_3$, 600 MHz).

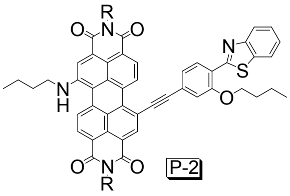

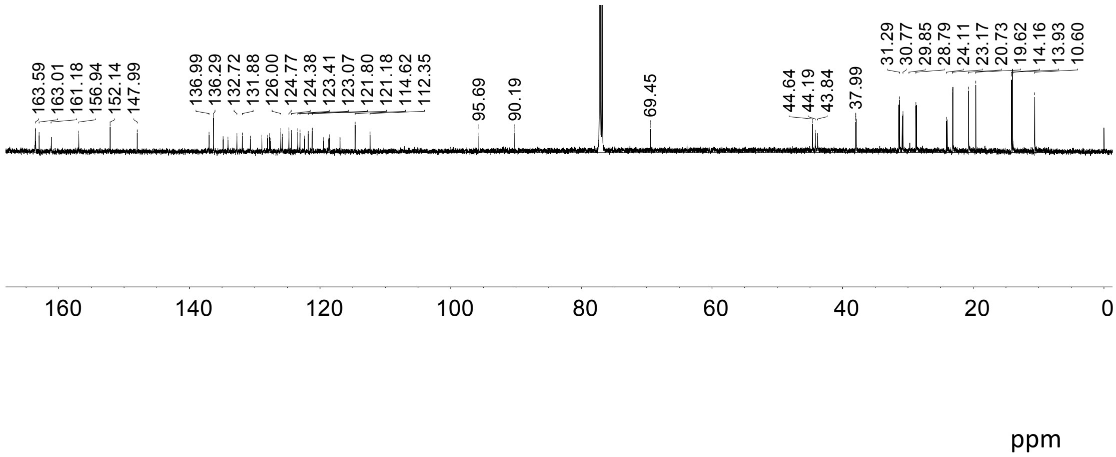

Fig S13. ^{13}C NMR of compound **P-2** ($CDCl_3$, 150 MHz).

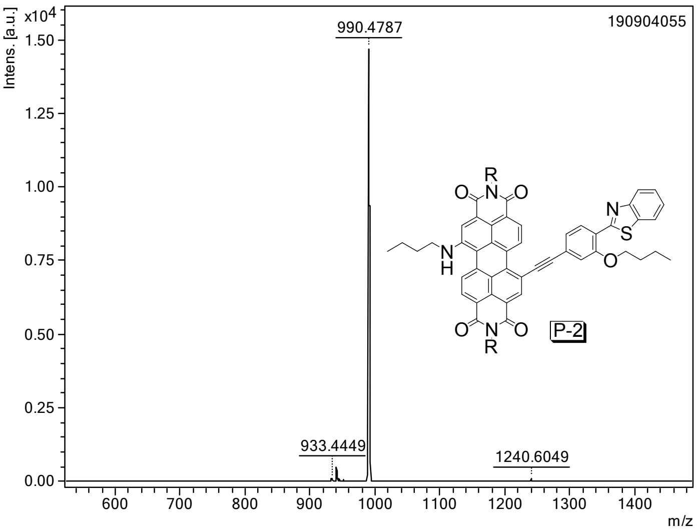

Fig S14. MALDI-HRMS of compound **P-2**

3. Photophysical Properties

Table S1 Photophysical Properties of **P-1**,**P-2**,7, **9** and **HBT** [a]

Comp.	Solvents	□$\lambda_{abs}{}^{b}$(nm)/A^{f}	ε^{c}($M^{-1}cm^{-1}$)	□$\lambda_{em}{}^{d}$(nm)	Stokes shift
**P-1**	EtOAc	652/0.237	23700	752	100
	CH_3CN	671/0.185	18500	-[p]	-[p]
	MeOH	647/0.159	15900	-[p]	-[p]
**P-2**	EtOAc	651/0.210	21000	756	105
	CH_3CN	686/0.145	14500	-[p]	-[p]
	MeOH	690/0.141	14100	-[p]	-[p]

**7**	EtOAc	518/0.820	82000	540	22
	CH_3CN	519/0.622	62200	541	22
	MeOH	481/0.187	18700	546	65
**9**	EtOAc	632/0.220	22000	713	81
	CH_3CN	638/0.224	22400	726	88
	MeOH	649/0.222	22200	732	83

[a]The excited wavelength for compound **P-1**, **P-2**, **7** and **9** were 620 nm, 620 nm, 500 nm and 600 nm respectively (1.0×10^{-5} M, 20 °C). [b]Absorption wavelength. [c] Molar extinction coefficient. [d] Fluorescence emission wavelength. [f] Absorbance. [p] Weak signal.

(The extinction coefficients is calculated according to the absorbance (A) of the maximum wavelength from the UV-vis spectrum and use Lambert's law to calculate the extinction coefficients (ε))

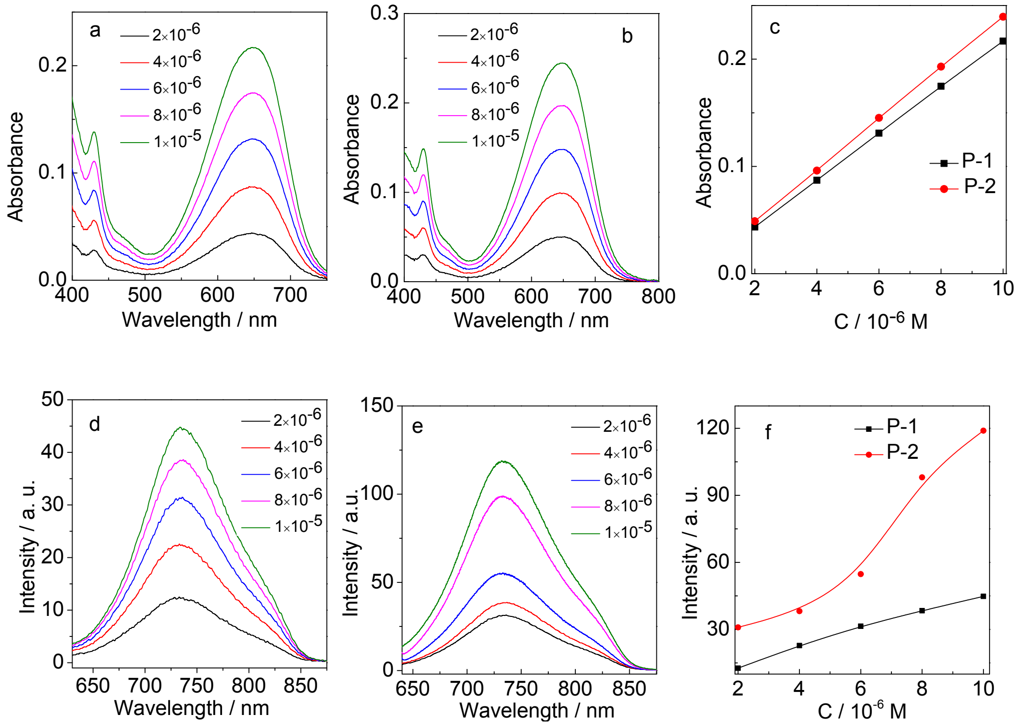

Fig S15. UV–Vis absorption spectra (a) **P-1**, (b) **P-2** and fluorescence emission spectra (d) **P-1**, (e) **P-2** at the different concentration. The dependence of (e) absorbance to concentration (f) fluorescence intensity to concentration for **P-1** and **P-2** in $PhCH_3$ at 20 °C.

3. Cyclic Voltammogram of P-1 and P-2

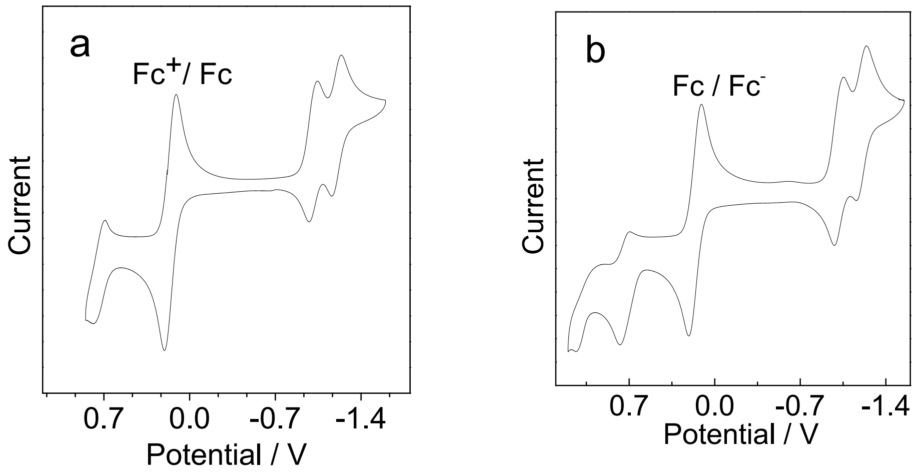

Figure S16 Cyclic voltammetry (a) **P-1** and (b) **P-2** in N_2-saturated solvents containing 0.10 M $Bu_4N[PF_6]$. Pt electrode as the counter electrode, glassy carbon as the electrode working electrode, Ferrocene (Fc/Fc^+) as the internal reference, and $Ag/AgNO_3$ couple as the reference electrode.

4. DFT calculation

Table S2. Selected parameters for the vertical excitation (UV-vis absorption and fluorescence emission) of the compounds. Electronic excitation energies (eV) and oscillator strengths (*f*), configurations of the low-lying excited states of **P-2** and its fluorescent precursors. Calculated by TDDFT//B3LYP/6-31G(d), based on the optimized ground state geometries ($PhCH_3$ was employed as solvent in all the calculation).

	Electronic transition[a]	Excitation energy	TDDFT/B3LYP/6-31G(d)		
			f[b]	Composition [c]	CI[d]
Absorption	$S_0 \rightarrow S_1$ [e]	1.78 eV (698 nm)	0.6036	$H \rightarrow L$	0.7079

	$S_0 \rightarrow S_7$	3.11 eV (399 nm)	0.2920	H → L+2	0.5506
				H → L+3	0.4283
	$S_0 \rightarrow S_{12}$	3.36 eV (369 nm)	0.1451	H-8 → L	0.5258
				H → L+4	0.3169
Emission	$S_1 \rightarrow S_0$	1.60 eV (774 nm)	0.5716	H → L	0.7099

[a] Only selected excited states were considered. The numbers in parentheses are the excitation energy in wavelength. [b] Oscillator strength. [c] H stands for HOMO and L stands for LUMO. Only the main configurations are presented. [d] Coefficient of the wavefunction for each excitations. The CI coefficients are in absolute values.

Compound **7** (DFT//B3LYP/6-31G(d))

Symbolic Z-matrix:

Charge = 0 Multiplicity = 1

C	-7.06545	-3.84523	-0.25588
C	-5.49653	-5.57641	0.56676
C	-6.35562	-2.8831	0.54184
C	-5.29113	-3.27463	1.41574
C	-4.91447	-4.65454	1.48865
C	-4.95657	-0.93272	2.07805
C	-5.99431	-0.52725	1.21089
C	-6.69858	-1.4915	0.45595
C	-7.74637	-1.07774	-0.40522
C	-8.52283	-2.03652	-1.07146
H	-9.39586	-1.68705	-1.61009
C	-6.44495	-7.40569	-1.38684
C	-5.51404	-7.84485	-0.42353
C	-5.02215	-6.93263	0.54385
H	-6.75443	-8.1039	-2.15504
C	-4.04768	-7.36259	1.47639
C	-3.52688	-6.45344	2.42152

H	-2.79327	-6.81752	3.13052
C	-8.26425	-3.39907	-0.96665
C	-3.93858	-5.13889	2.42321
H	-3.51766	-4.46444	3.15682
C	-4.61896	-2.26292	2.18027
C	-8.09398	0.34237	-0.53703
C	-6.32052	0.90192	1.10661
O	-5.70622	1.77277	1.7688
O	-9.01064	0.74568	-1.29322
C	-5.03403	-9.23127	-0.46539
C	-3.56156	-8.74876	1.47345
N	-4.09311	-9.61318	0.50395
O	-2.70564	-9.15851	2.29463
O	-5.43989	-10.05421	-1.3218
C	-3.61015	-11.00677	0.48458
H	-4.40139	-11.63927	0.08624
H	-2.7267	-11.09619	-0.15561
H	-3.3412	-11.29165	1.50016
N	-7.3559	1.25778	0.22618
C	-7.71174	2.68244	0.08811
H	-7.58345	2.9959	-0.95036
H	-8.75825	2.82975	0.36404
H	-7.05756	3.25131	0.74466
C	-6.53157	-5.17348	-0.34291
C	-6.93553	-6.1185	-1.35375
H	-4.38595	-0.20448	2.63525
H	-8.97459	-3.96167	-1.55427
H	-7.61199	-5.80555	-2.12146

H	-3.82274	-2.5699	2.82577

Compound **9** (DFT//B3LYP/6-31G(d)/GENECP)

Symbolic Z-matrix:

Charge = 0 Multiplicity = 1

C	-0.5301	-1.56405	-0.0631
C	1.52518	-0.20505	-0.02798
C	-1.31702	-0.36396	-0.09001
C	-0.70158	0.93115	0.03076
C	0.75264	0.99174	-0.18474
C	-2.96013	1.88867	0.21456
C	-3.54358	0.67264	-0.09001
C	-2.73273	-0.47732	-0.22679
C	-3.33207	-1.73045	-0.49802
C	-2.5328	-2.85706	-0.61616
H	-2.99639	-3.80518	-0.86707
C	3.12663	-2.45446	0.46954
C	3.72667	-1.2876	0.06341
C	2.9421	-0.12602	-0.15894
H	3.75819	-3.30813	0.69879
C	3.56661	1.0812	-0.53537
C	2.79284	2.20397	-0.78552
H	3.27544	3.11798	-1.11324
C	-1.15808	-2.77718	-0.3771
C	1.4078	2.15552	-0.60461
H	0.83695	3.04762	-0.81421
C	-1.56756	2.00716	0.2807
C	-4.79434	-1.8432	-0.6681

C	-5.0178	0.59408	-0.22298
O	-5.74021	1.57534	-0.08574
O	-5.35742	-2.90168	-0.92778
C	5.20473	-1.25114	-0.0803
C	5.03753	1.14997	-0.69853
N	5.76536	-0.02396	-0.45607
O	5.62208	2.17341	-1.03274
O	5.90352	-2.23848	0.11875
C	7.22122	0.06661	-0.61996
H	7.64285	-0.90832	-0.38792
H	7.46086	0.3519	-1.64708
H	7.62199	0.8275	0.05365
N	-5.54803	-0.66905	-0.51813
C	-7.00028	-0.80275	-0.68047
H	-7.22641	-1.18422	-1.67892
H	-7.39212	-1.50801	0.05637
H	-7.44226	0.18041	-0.53821
N	1.23286	-3.73906	1.15659
H	1.96415	-4.41734	1.32531
C	0.1634	-3.78629	2.15386
H	0.40598	-4.56412	2.88178
H	0.07585	-2.82816	2.67849
H	-0.81153	-4.01894	1.71201
C	0.90067	-1.47011	0.22101
C	1.71784	-2.56031	0.61833
H	-3.59823	2.74731	0.38364
H	-0.55699	-3.67594	-0.44663
Br	-0.97086	3.79962	0.88302

Compound **P-1** (DFT//B3LYP/6-31G(d))

Symbolic Z-matrix:

Charge = 0 Multiplicity = 1

C	-7.06545	-3.84523	-0.25588
C	-5.49653	-5.57641	0.56676
C	-6.35562	-2.8831	0.54184
C	-5.29113	-3.27463	1.41574
C	-4.91447	-4.65454	1.48865
C	-4.95657	-0.93272	2.07805
C	-5.99431	-0.52725	1.21089
C	-6.69858	-1.4915	0.45595
C	-7.74637	-1.07774	-0.40522
C	-8.52283	-2.03652	-1.07146
H	-9.39586	-1.68705	-1.61009
C	-6.44495	-7.40569	-1.38684
C	-5.51404	-7.84485	-0.42353
C	-5.02215	-6.93263	0.54385
H	-6.75443	-8.1039	-2.15504
C	-4.04768	-7.36259	1.47639
C	-3.52688	-6.45344	2.42152
H	-2.79327	-6.81752	3.13052
C	-8.26425	-3.39907	-0.96665
C	-3.93858	-5.13889	2.42321
H	-3.51766	-4.46444	3.15682
C	-4.61896	-2.26292	2.18027
C	-8.09398	0.34237	-0.53703
C	-6.32052	0.90192	1.10661

O	-5.70622	1.77277	1.7688
O	-9.01064	0.74568	-1.29322
C	-5.03403	-9.23127	-0.46539
C	-3.56156	-8.74876	1.47345
N	-4.09311	-9.61318	0.50395
O	-2.70564	-9.15851	2.29463
O	-5.43989	-10.05421	-1.3218
C	-3.61015	-11.00677	0.48458
H	-4.40139	-11.63927	0.08624
H	-2.7267	-11.09619	-0.15561
H	-3.3412	-11.29165	1.50016
N	-7.3559	1.25778	0.22618
C	-7.71174	2.68244	0.08811
H	-7.58345	2.9959	-0.95036
H	-8.75825	2.82975	0.36404
H	-7.05756	3.25131	0.74466
C	-6.53157	-5.17348	-0.34291
C	-6.93553	-6.1185	-1.35375
H	-4.38595	-0.20448	2.63525
H	-8.97459	-3.96167	-1.55427
H	-7.61199	-5.80555	-2.12146
H	-3.82274	-2.5699	2.82577

Compound **P-2** (DFT//B3LYP/6-31G(d))

Symbolic Z-matrix:

Charge = 0 Multiplicity = 1

C	-7.06545	-3.84523	-0.25588
C	-5.49653	-5.57641	0.56676

C	-6.35562	-2.8831	0.54184
C	-5.29113	-3.27463	1.41574
C	-4.91447	-4.65454	1.48865
C	-4.95657	-0.93272	2.07805
C	-5.99431	-0.52725	1.21089
C	-6.69858	-1.4915	0.45595
C	-7.74637	-1.07774	-0.40522
C	-8.52283	-2.03652	-1.07146
H	-9.39586	-1.68705	-1.61009
C	-6.44495	-7.40569	-1.38684
C	-5.51404	-7.84485	-0.42353
C	-5.02215	-6.93263	0.54385
H	-6.75443	-8.1039	-2.15504
C	-4.04768	-7.36259	1.47639
C	-3.52688	-6.45344	2.42152
H	-2.79327	-6.81752	3.13052
C	-8.26425	-3.39907	-0.96665
C	-3.93858	-5.13889	2.42321
H	-3.51766	-4.46444	3.15682
C	-4.61896	-2.26292	2.18027
C	-8.09398	0.34237	-0.53703
C	-6.32052	0.90192	1.10661
O	-5.70622	1.77277	1.7688
O	-9.01064	0.74568	-1.29322
C	-5.03403	-9.23127	-0.46539
C	-3.56156	-8.74876	1.47345
N	-4.09311	-9.61318	0.50395
O	-2.70564	-9.15851	2.29463

O	-5.43989	-10.05421	-1.3218
C	-3.61015	-11.00677	0.48458
H	-4.40139	-11.63927	0.08624
H	-2.7267	-11.09619	-0.15561
H	-3.3412	-11.29165	1.50016
N	-7.3559	1.25778	0.22618
C	-7.71174	2.68244	0.08811
H	-7.58345	2.9959	-0.95036
H	-8.75825	2.82975	0.36404
H	-7.05756	3.25131	0.74466
C	-6.53157	-5.17348	-0.34291
C	-6.93553	-6.1185	-1.35375
H	-4.38595	-0.20448	2.63525
H	-8.97459	-3.96167	-1.55427
H	-7.61199	-5.80555	-2.12146
H	-3.82274	-2.5699	2.82577
